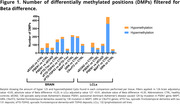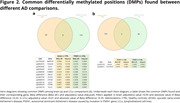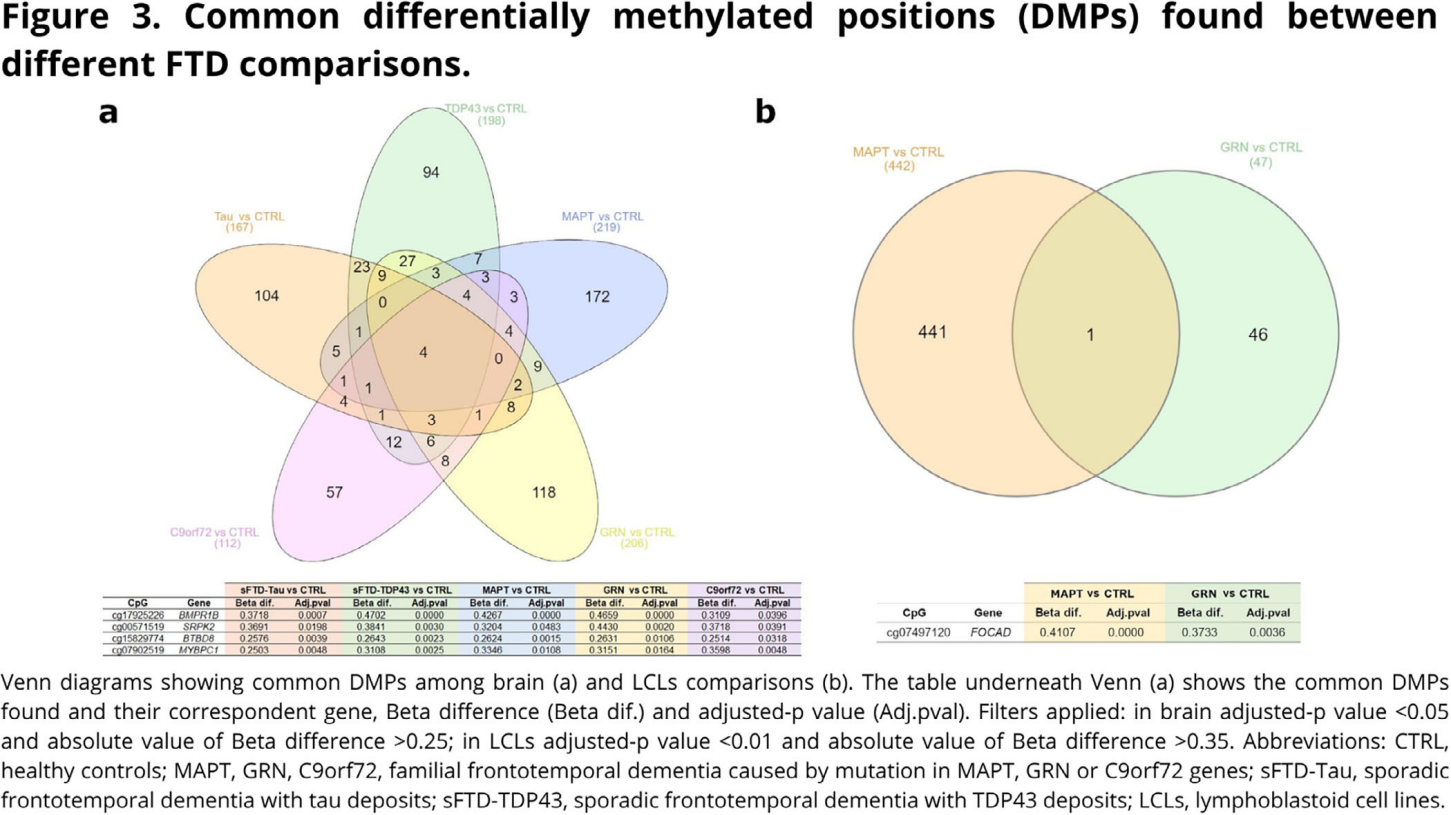# Genome‐wide DNA methylation in early‐onset dementias brain tissue and lymphoblastoid cell lines

**DOI:** 10.1002/alz.088732

**Published:** 2025-01-03

**Authors:** Aina Comas‐Albertí, Oscar Ramos‐Campoy, Laura Fort‐Aznar, David Hervás‐Marín, Sergi Borrego‐Écija, Bea Bosch, Juan Sandoval, Fermin Moreno, Guadalupe Fernandez‐Villullas, Laura Molina, Mircea Balasa, Albert Lladó, Raquel Sanchez‐Valle, Anna Antonell

**Affiliations:** ^1^ Alzheimer’s disease and other cognitive disorders Unit. Hospital Clínic de Barcelona; FRCB‐IDIBAPS; University of Barcelona, Barcelona Spain; ^2^ Universitat Politècnica de València, Valencia Spain; ^3^ Epigenomics core facility, Health Research Institute La Fe, Valencia Spain; ^4^ Hospital Universitario Donostia, San Sebastián Spain; ^5^ Neuroscience Area, Biodonostia Health Research Institute, Gipuzkoa, San Sebastian Spain; ^6^ Neurological Tissue Bank of the Biobank‐IDIBAPS‐Hospital Clínic, Barcelona Spain; ^7^ Hospital Clínic de Barcelona ‐ Fundació de Recerca Clínic Barcelona – IDIBAPS ‐ University of Barcelona, Barcelona, Catalonia Spain

## Abstract

**Background:**

Epigenetic mechanisms as a potential underlying pathogenic mechanism of neurodegenerative diseases have been the scope of several studies performed so far. However, there is a gap in analyzing different forms of early‐onset dementia to minimize the effect of aging and the use of Lymphoblastoid cell lines (LCLs) as a possible disease model for earlier clinical phases.

**Method:**

We performed a genome‐wide DNA methylation analysis in 64 samples (from prefrontal cortex and lymphoblastoid cell lines) from Alzheimer’s Disease (AD) and Frontotemporal dementia (FTD) using the Illumina Infinium MethylationEPIC V2.0 array. The studied cohort included sporadic early‐onset (sEOAD, sFTD‐TP43, sFTD‐Tau) and genetic subgroups of AD (*PSEN1*) and FTD (*MAPT, GRN, C9orf72*), with n = 5 subjects/group. We analyzed the differentially methylated positions (DMPs) using the Beta regression model, with age and sex as covariates, and all p‐values adjusted by False Discovery Rate (FDR). Venn diagrams to visualize common genes between pairwise comparisons and heatmaps were performed to further explore the most important DMPs. Elastic Net logistic regression was used to obtain epigenetic diagnostic signatures. We also performed a correlation analysis of DNA methylation levels with Clariom D array gene expression data for the same cohort.

**Result:**

Results showed hypermethylation in patients’ groups as the most frequent finding in both tissues studied (Fig. 1). We identified common DMPs when comparing patients with healthy controls (CTRL) for each respective disease (Fig. 2, 3). Biological significance analysis revealed common pathways altered in AD and FTD affecting neuron development, metabolism, signal transduction and immune system pathways. These alterations were also found in LCLs, even some related to neuron development. We obtained diagnostic signatures to differentiate patients from CTRL. In the brain, CpG methylation presented an inverse correlation with gene expression, while in LCLs we observed mainly a positive correlation.

**Conclusion:**

This study enhances our understanding about the biological pathways that are associated with neurodegeneration, describes differential methylation patterns, and suggests LCLs are a potential cell model for studying neurodegenerative diseases in early clinical phases.